# Degradation Behavior of Biodegradable Man-Made Fibers in Natural Soil and in Compost

**DOI:** 10.3390/polym15132959

**Published:** 2023-07-06

**Authors:** Pia Borelbach, Rodion Kopitzky, Jörg Dahringer, Patrick Gutmann

**Affiliations:** 1 Fraunhofer Institut für Umwelt-, Sicherheits- und Energietechnik UMSICHT, 46047 Oberhausen, Germany; 2 Indorama Ventures Fibers Germany GmbH, 86399 Bobingen, Germany

**Keywords:** biodegradation, fiber, soil, compost, PHA, PLA, PBS

## Abstract

In open environment applications, fibers are increasingly being used that are expected to biodegrade in the soil after their desired service life. Biodegradable polymer fibers are a versatile alternative to natural fibers. In this study, the degradation behavior of fibers made from polylactic acid (PLA) and a polyhydroxy alkanoate (PHA) blend with PLA, as well as a bicomponent fiber (BICO) made from polybutylene succinate (PBS) and PLA, was investigated. The fibers were stored in topsoil at 23 °C for 12 weeks. In addition, fibers were stored in compost at 58 °C for 4 weeks to investigate the degradation behavior in an industrial composting plant. Reference materials were also stored without substrate under the same temperatures and humidity conditions. Samples were taken regularly, and mechanical testing, scanning electron microscopy (SEM), gel permeation chromatography (GPC), differential scanning calorimetry (DSC), and infrared spectroscopy (IR) were used to study the degradation of the fibers. After 12 weeks in soil at ambient temperatures, the PLA and BICO fibers showed no degradation. The PHA fibers showed cracks in SEM, a decrease in molecular weight, and changes in the IR spectrum. No evidence of biological influence (bacteria or fungi) was found. Under industrial composting conditions, all fibers showed a decrease in strength and molecular weight. For the BICO and the PHA fibers, the SEM images show significant changes. Especially in the PHA fibers, fungal mycelia can be seen. The studies provide a better insight into the processes involved in the degradation behavior under different environmental conditions.

## 1. Introduction

Due to their good material properties and flexible processability, plastics are an important part of everyday life. On the contrary, plastics are increasingly viewed critically in society—especially because of the discussions about plastic waste in the environment [1,2]. Consumers are asking more and more for sustainable solutions. The industry is therefore rethinking the use of more sustainable alternatives, such as bio-based and biodegradable plastics [3,4,5]. During their service life, the materials must fully meet the required technical properties. A targeted adjustment of degradability is therefore of high technical and economic interest for many manufacturing and processing companies. Textiles, for example, are used in many applications where targeted control of biodegradability is useful, especially in horticultural and agricultural applications and as geotextiles.

Fibers and textiles are biodegradable if they can be decomposed aerobically by microorganisms into carbon dioxide, water, mineral salts, and biomass, or anaerobically into carbon dioxide, methane, mineral salts, and biomass [6,7]. This process is highly dependent on the environmental conditions, the microorganisms present, and the material properties, which means that no generally valid statement on degradability can be made that applies to all environmental compartments [6,8,9]. Even before visible degradation, environmental conditions affect the material and cause “environmental aging”. The degradation of polyesters basically begins with the hydrolysis of the polymer chains into smaller fragments, which are subsequently taken up by microorganisms through the cell membrane and further processed in the metabolism [10].

Hydrolysis is accelerated by enzymatic reactions. The polymer-cleaving enzymes are excreted from the cells by the microorganisms as exoenzymes and attack the polymer surface. Abiotic processes such as purely chemical hydrolysis may also be involved [9].

Due to their processing properties, only some of the available biodegradable polymers are suitable for the melt spinning process. Filament extrusion in the melt spinning process is limited to polymers that are thermally stable at the processing temperatures and thus do not degrade in the process. Polylactic acid (PLA) fiber processing is well known [11,12] and PLA fibers are commercially available, e.g., from Indorama Ventures Fibers Germany GmbH (former: Trevira GmbH) [13]. Melt spun fibers made of polybutylene adipate-co-terephthalate (PBAT) [14,15], polybutylene succinate-co-butylene terephthalate (PBST) [16,17], polybutylene succinate (PBS) [17,18,19,20], polyhydroxybutyrate (PHB) [21,22,23], PBS/PBAT blends [24], PLA/PBS blends [25,26] and PLA/PHB blends [27] have been successfully produced in various research projects. The polyhydroxy alkanoate (PHA) poly(3-hydroxybutyrate-co-3-hydroxy-valerate) (PHBV) is processable into fibers when used as a bicomponent fiber with PLA sheath and PHBV core [28].

The biodegradation of PLA under industrial composting conditions according to DIN EN ISO 14855-1 has been studied by many research groups. In this method, the aerobic biodegradation of the sample is determined via the generation of carbon dioxide (CO_2_) under controlled composting conditions (58 °C ± 2 °C, compost humidity 50–55%) in a closed system (e.g., respirometer). For example, Kale et al. [29] investigated the biodegradation performance of cut PLA bottles. The pieces in the size of 10 × 10 mm^2^ degraded to 81% ± 10% in 58 days. Rudeekit et al. [30] found 86% degradation of plates in size 20 × 20 × 0.3 mm^3^ after 120 days, and Stloukal et al. [31] found 75% after 90 days with 2 mm pieces of PLA. Among others, Kijchavengkul et al. [32] have dealt with the degradation of PBAT. PBAT film pieces with a thickness of about 38 µm degrade in manure compost to 67% after 45 days. For PBST, no data on degradation under composting conditions were found. Gil-Castell et al. [33] state a disintegration of 25% for poly(3-hydroxybutyrate-co-3-hydroxyhexanoate) (PHBH) after 90 days in compost, measured by mass loss. Anstey et al. [34] have tested PBS powder according to ASTM 6400 and found a biodegradation rate of 90% after 160 days. Yang et al. [35] tested PBS film pieces (50 µm thickness) and powder (200 µm), among other substances, and found a faster degradation at the early and later stages of the powder (18% compared to 12% after 45 days). The same influence was found for PLA (55 µm and 200 µm) but not for polybutylene succinat-co-adipate (PBSA). The researchers conclude that with a larger available surface area, the degradation rate increases, and for the more easily degrading PBSA, the surface area of the film became practically as large as that of the powder.

In contrast to the industrial composting process at higher temperatures with a thermophilic phase, it is well known that PLA degrades very slowly in compost and soil under ambient conditions (<30 °C) [36,37,38]. For example, Palsikowski et al. [39] measured a degradation of 16% after 180 days in soil at 25 °C. In soil at room temperature, 34% of the polymer PHBV was degraded [40]. Gómez et al. [41] degraded PHA in compost at 20 °C. After 660 days, 69% of the initial carbon was lost (in relation to the control sample of cellulose paper: 93%). PHB-copolymer degrades after 120 days to 90% in natural soil at 25 °C [42], and PBAT to 21% after 180 days [39]. In experiments by Debuissy et al. [43], PBS in soil at 30 °C degraded to approx. 60% after 180 days.

In the degradation tests described above, most biodegradable plastics were used as sheets, films, or powders. An investigation of fibers in comparison with films was carried out by Rudnik et al. [44]. The fibers showed slower degradation than the films, but the fibers were placed in soil and compost as bundles in nets and thus may not have had optimal contact with the substrate, and the surface area interacting with the biotic environment may have been effectively smaller than the total fiber surface. In general, most studies on the biodegradation of biodegradable polymers do not consider the influence of the sample form of the polymer, and thus comprehensive quantitative descriptions of the influence of, for example, the aspect ratio on degradation are not available.

It is well known that hydrolysable polymers such as polyesters, polycarbonates, and polyamides show chemical degradation by hydrolysis of the surface or by bulk degradation depending on the hydrolysis velocity constant, the diffusion constant, and the thickness of the sample [45,46,47]. Especially in the case of polyesters, this may have an impact on overall degradation behavior, as indicated by an erosion number in the range of 1 [45]. Therefore, the biological degradation of orthoesters with an erosion number of >>1 is limited by the velocity of the biological transformation (transport through cell walls and degradation in the cell), as hydrolysis to monomers and oligomers is fast. Contrary to this, polyamide biological degradation is limited by the effectiveness of the polymer chain degrading (exo)enzymes of the bacteria, which cut of the polymer chain into cell wall-permeating oligomers due to their extremely low velocity of chemical hydrolysis. Polyesters lie between these two extremes, and the rate of chemical hydrolysis of the surface may be of the same order of magnitude as the enzymatic cleavage of the chain. As powders have a greater surface-to-volume ratio than fibers, and fibers have a greater surface-to-volume ratio than films, the sample form may influence biodegradation, and chemical hydrolysis should not be neglected a priori, especially in environments with pH values slightly below or above 7, as they are common in soil or compost, accelerating chemical hydrolysis [48].

To select biodegradable fibers for use in environmental applications, for instance, geotextiles, knowledge about their degradation behavior in the use phase and afterwards is necessary. Up until now, there has been insufficient data in the literature.

The aim of the presented research was to increase knowledge of the degradation behavior of the three different fibers. For the behavior during the service life, aging in soil at room temperature was chosen. One of the end-of-life options is industrial composting, which was therefore also investigated. To ensure good contact of the fibers with the substrate, a self-constructed fiber sample holder was used.

## 2. Materials and Methods

### 2.1. Materials

Indorama Ventures Fibers Germany GmbH (former: Trevira GmbH, Bobingen, Germany) provided three different crimped fibers prior to cutting for the degradation tests:

- type T400, fineness 6.7 dtex, material: PLA (density ~ 1.25 g cm^−3^ → approximately d = 26 µm)
- type T453, fineness 2.2 dtex, bicomponent (BICO) fiber, core material: PLA, shell material: PBS (density ~ 1.25 g cm^−3^ → approximately d = 15 µm),
- type V400, fineness 1.7 dtex, material: PHA/PLA blend (density ~ 1.25 g cm^−3^ → approximately d = 13 µm).

### 2.2. Sample Preparation

**Figure 1:**
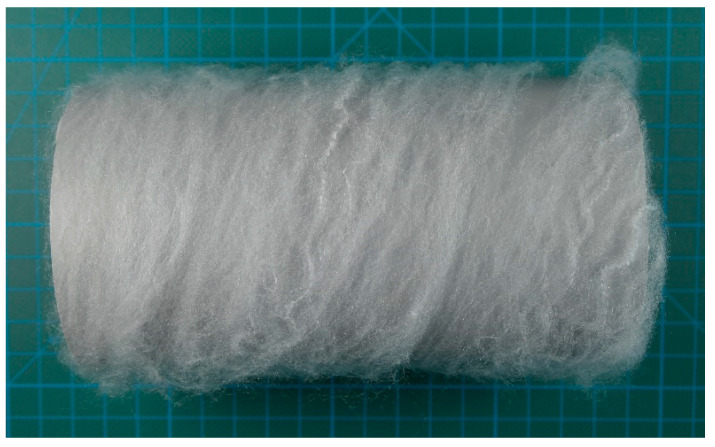
Sample holder with fanned PLA fibers.

To test the degradation of micron-sized (diameter) fibers in soil or compost, the fibers must have good contact with the substrate on the one hand, and on the other hand, it must be possible to remove the fibers from the substrate without causing additional damage. If the fibers are placed directly into the substrate, removal is difficult, especially in the case of fibers that have already experienced degradation. In mesh bags, contact with the substrate is not guaranteed. Therefore, in this study, sample holders for the fibers were devised. Uncut fibers, i.e., fiber bundles, were wound by hand onto polypropylene pipes with a diameter of 75 mm and a length of about 140 mm (Figure 1). In the process, the fibers were fanned out to achieve a thin layer on the holder. In addition, care was taken not to generate any tension in the fibers. The ends of the fiber bundles were fixed to the inside of the pipe with adhesive tape.

### 2.3. Natural Soil Burial Test

Topsoil (obtained by Baustoffe Ruhr GmbH, Essen, Germany) was sieved using a mesh size of 3 mm to obtain a homogeneous mix and then moistened with tap water. To determine the pH of the soil, 20 g of soil was mixed with 200 mL of a 0.01-molar CaCl_2_ solution. The suspension was stirred for 1 h and then allowed to stand for about 1 h. After this, the pH value of the suspension is measured. The soil pH value measured was 7.4. Moisture content was measured with the moisture analyzer MLS 50-3 (KERN & SOHN, Balingen, Germany) and was approximately 22 wt%. Four sample holders, each with the same fibers, were completely buried in the prepared soil in weighted plastic boxes provided with air holes for ventilation. The plastic boxes were stored for a total of 12 weeks at 23 °C and 50% relative humidity. The evaporated water was supplemented weekly according to the weight loss. A sample of each fiber was taken at 14, 28, 56, and 84 days, respectively. The removed fibers were carefully mechanically cleaned with a fine paint brush, minimizing mechanical impact, under a trickle of running tap water, and stored for 24 h at 23 °C and 50% humidity to dry.

In parallel, each material was wound on sample holders, packed in a plastic box without soil, and stored for 12 weeks at 23 °C and 50% humidity. After 4 and 12 weeks, the same procedure was followed as for the samples stored in the soil.

### 2.4. Compost Burial Test

Compost (obtained by waste disposal center Asdonkshof, Kamp-Lintfort, Germany) was sieved using a mesh size of 5 mm. Moisture content was set to 53 wt% with tap water, and compost pH measured using the method described in chapter 2.3 was 7.1. Four sample holders, each with the same fibers, were completely buried in the prepared compost in plastic boxes provided with air holes for ventilation. Each plastic box was weighted. The plastic boxes were stored for a total of 4 weeks in a climate chamber at 58 °C and 70% relative humidity. Once a week, the evaporated water was supplemented. One sample of each fiber was taken at 3, 7, 14, and 28 days. The removed fibers were carefully cleaned with a fine paint brush under a trickle of running tap water and stored for 24 h at 23 °C and 50% humidity to dry.

In parallel, each material was packed in a sample holder in a plastic box without compost and stored for 4 weeks in the climate chamber at 58 °C and 70% relative humidity. After 4 weeks, the same procedure was followed as for the samples stored in compost.

### 2.5. Mechanical Tests

Linear density measurements and tensile tests were determined with a Favigraph M testing machine (Textechno Herbert Stein GmbH & Co. KG, Mönchengladbach, Germany) except for the PLA reference fiber, which was measured with a Favimat (Textechno Herbert Stein GmbH & Co. KG, Mönchengladbach, Germany). Both systems work with a pneumatic clamping system and a 100 cN load cell. The Favigraph works with a transfer clamp between the two measuring systems, into which the appropriately pre-tensioned fiber is manually inserted. First, the linear density of the fiber is determined using the vibration method according to ASTM D1577. This test method determines the resonance frequency of the sample at a constant gauge length and known pre-tension. The transfer clamp then moves to the tensile test section and places the fiber in the measuring and draw-off clamp [49]. For each determination, 38 to 50 single fibers were measured with a gauge length of 50 mm at a speed of 40 mm min^−1^.

### 2.6. Gel Permeation Chromatography (GPC)

A GPC instrument (Agilent 1260 Series, PSS GmbH, Mainz, Germany) was applied to determine the molecular weights of the fiber samples. A portion of each fiber sample was weighed (9.0 mg–9.5 mg) and dissolved in 1,1,1,3,3,3-hexafluoro-2-propanol (HFIP) (3 mL) containing potassium trifluoroacetate (KTFAc) (0.05 mol L^−1^). The completely dissolved sample was then filtered through a polytetrafluoroethylene (PTFE) membrane filter (pore size: 0.25 µm). Each sample (100 µL) was injected into the mobile phase with an isocratic pump (G1310B, Agilent Series 1260) and transported through the GPC columns (flow rate: 1 mL min^−1^). The column set includes three PSS-PFG columns for fluorinated solvents connected in series (pore size: 1000 Å, 300 Å, and 100 Å, respectively; particle size: 7 µm; column size: 8.0 mm × 300 mm; PSS GmbH, Mainz, Germany). Detection was performed with a refractive index (RI) detector (G1362A, Agilent 1260 Series). A narrow molecular weight distribution polymethylmethacrylate (PMMA) standard (PSS Mainz, Germany) was used as calibration. The elution curves were examined with the WinGPC^®^ UniChrom (Version 8.31) Software (PSS, Mainz, Germany).

### 2.7. Scanning Electron Microscope (SEM)

Scanning electron microscope examinations were carried out with a SEM (Vega3, Tescan GmbH, Dortmund, Germany) with an acceleration voltage of 20 kV and a secondary electron detector (SED). The fiber surfaces were sputtered with gold using a sputter-coater (Cressington 108, Cressington, Dortmund, Germany) for 180 s at 30 mA.

### 2.8. Attenuated Total Reflectance Fourier Transform Infrared Spectrometer (ATR-FT-IR)

The infrared spectra from the fiber samples were obtained using an infrared spectrometer (Vertex 70, Bruker GmbH, Ettlingen, Germany) with a Golden Gate ATR (Golden Gate, Specac Ltd., Orpington, UK). 16 spectra with a resolution of 4 cm^−1^ were coadded. Evaluation of spectra was performed with Bruker OPUS Software Version 7.8.44.

### 2.9. Differential Scanning Calorimetry (DSC)

Thermo-analytical properties of fiber samples were measured via differential scanning calorimetry (DSC 204 F1 Phoenix^®^, Netzsch Gerätebau GmbH, Selb, Germany). The samples were defibrillated, and a small ball was formed and flattened to fit the bottom of the aluminum crucible. In order to ensure the discharge of any gases that may be produced, the lid was provided with a hole on the convex side. With the concave side facing out, it is used to lightly press the sample inside to improve the contact of the fibers with the bottom of the crucible. The DSC cell was constantly purged with nitrogen (flow rate: 20 mL min^−1^). Each sample was measured using the same temperature profile (first heating: 25 °C to 200 °C; holding time: 3 min; cooling: 200 °C to 0 °C; holding time: 15 min; second heating: 0 °C to 250 °C; heating and cooling rates: 10 K min^−1^). Evaluation of thermograms was performed with Netzsch Proteus—Thermal Analysis—Software Version 8.0.3.

## 3. Results

### 3.1. Natural Soil/Ambient Temperature

#### 3.1.1. Natural Soil: Microscopy

**Figure 2:**
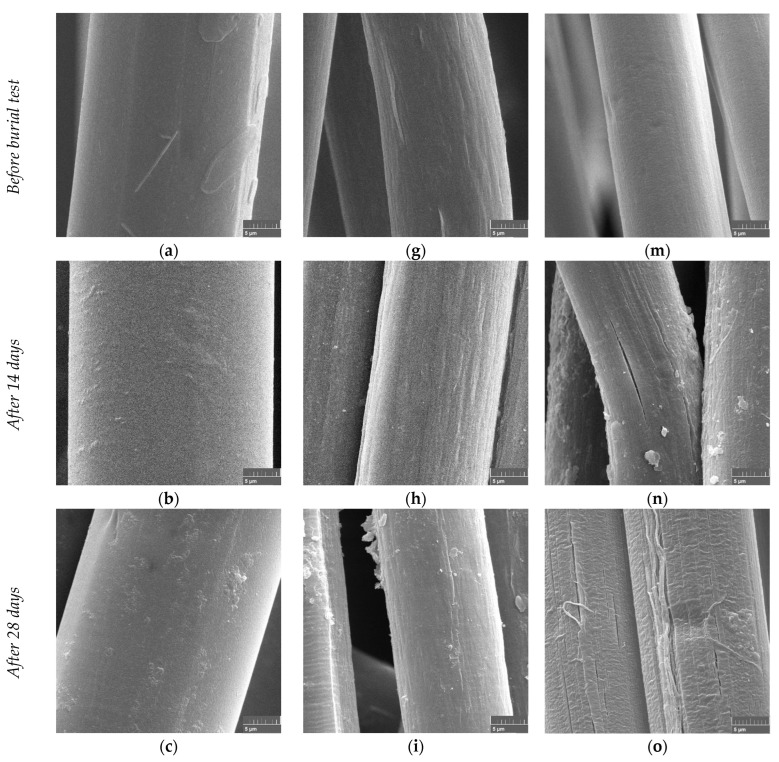

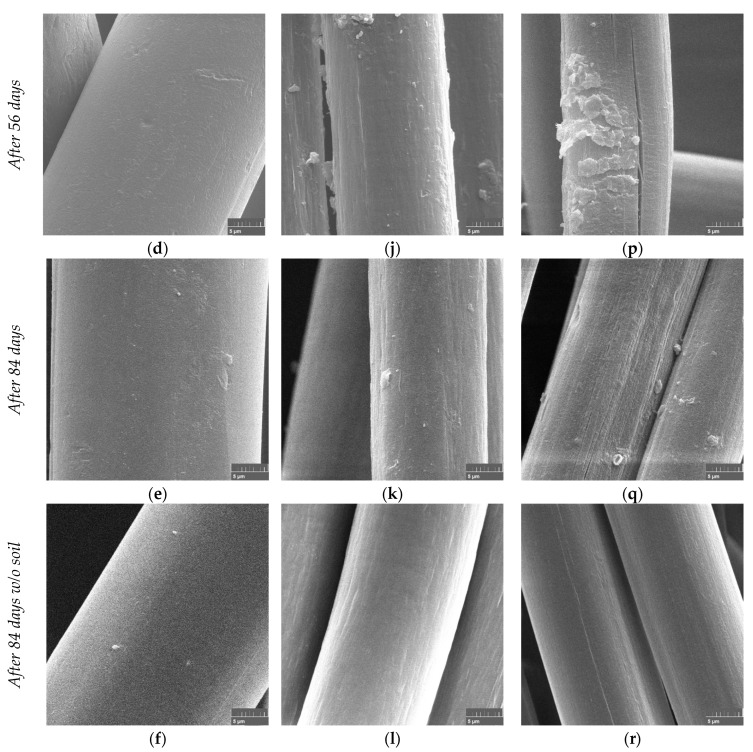
SEM micrographs of fibers buried in soil after 0, 14, 28, 56, and 84 days: (**a**–**f**) PLA, (**g**–**l**) PBS/PLA BICO fiber, (**m**–**r**) PHA/PLA blend fiber; the size bar indicating 5 µm is placed at the bottom right of each image.

The PLA fibers taken from the soil after 14, 28, 56, and 84 days remained bright. For the PBS/PLA BICO fibers, yellowish discoloration appeared after 28 days, even after careful cleaning. The PHA/PLA blend fibers showed yellowish discoloration after 14 days. SEM images were obtained for a more detailed investigation of the optical change of the fibers. Figure 2 shows SEM images of the fibers during their aging in the soil at ambient temperatures.

The SEM micrographs of the surface of the PLA and PBS/PLA BICO fibers show practically no change with aging in the soil.

The surfaces of the PLA fibers remain essentially unchanged and show no changes indicating physicochemical degradation or biological attack. In addition, no adhering particles can be seen in the images, indicating no strong polarity of the surface for interacting with polar minerals or polyelectrolytic humic substances. The surface of the PBS/PLA BICO fiber already shows some roughness at the beginning, which does not increase significantly during the 12 weeks of storage in the topsoil. The adhesions, which are visible in the images after 28 and 56 days (Figure 2i–j), can be an indication of a slight change in the surface polarity, which can lead to agglomeration and adhesion of polyelectrolytic humic substances. In contrast to PLA and PBS/PLA BICO fibers, individual PHA/PLA blend fibers show first crazes already after two weeks of storage in the soil (Figure 2l), which become more pronounced with time (Figure 2n). These crazes and cracks do not belong to a direct biological attack, as no patterns can be seen that indicate the presence of fungi and bacteria, such as fungal hyphae and bacterial colonization as shown in the biodegradation in soil after 6 weeks by Zumstein et al. for the PBAT polyester [10], or grooves formed by the morphology of filamentous microorganisms mentioned by Jarerat et al. [50] in the case of PLA degradation in a solid culture, or spherical holes formed in the amorphous and crystalline parts of the PHB polymer illustrated by Tokiwa and Calabia [50,51,52]. Further on, the crazes and cracks seen, especially in Figure 2p, are reminiscent of the appearance of crazes and cracks that can occur due to swelling caused by water absorption [53]. In addition, some surface roughness beginning after two weeks (Figure 2n) and more clearly after 4 weeks (Figure 2o) with delamination of fiber fragments can be seen. The surface of the aged PHA/PLA blend fiber looks, in general, rougher and shows more adhering particles than the PLA or PBS/PLA BICO fiber, indicating the greater overall environmental aging of the surface. In contrast, the fibers of all three materials stored for 12 weeks at 23 °C and 50% relative humidity without natural soil show no visible change over time.

#### 3.1.2. Natural Soil: Mechanics/Molecular Weight/FT-IR/DSC Characterization

**Figure 3:**
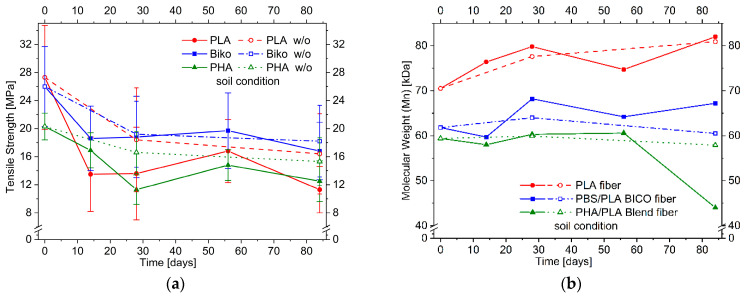
(**a**) Effect of soil burial duration up to 84 days at 23 °C on the tensile strength and (**b**) molecular weight (M_n_) of PLA, PBS/PLA BICO, and PHA/PLA blend fibers; open symbols/dashed lines indicate storage under the same conditions but without soil.

The tensile strength of PLA, PBS/PLA BICO, and PHA/PLA blend fibers as a function of natural soil burial time is shown in Figure 3a. The tensile strength of the fibers tends to decrease over time, but even though a decrease in strength can be observed for all three fibers after 2 weeks, this decrease does not continue over time. In contrast, the strength increases again after 8 weeks for all fibers stored in soil. Even for the PHA/PLA blend fibers, which were expected to degrade fastest, the strength after 12 weeks is higher than after 4 weeks. Fibers that were stored without soil but otherwise identically and measured after 2 and 12 weeks show a trend towards a decrease in strength over time, but due to the size of the error in the fibers stored in soil, there is no unambiguity here either.

Significant changes in the sum of heats of fusion obtained from the first heating in the DSC (Tables S1, S3 and S5, supporting information), indicating a change in crystallinity and therefore a change in strength, are not evident with the exception of the PHA/PLA blend fibers after 8 and 12 weeks. Therefore, one could expect a slight decrease in strength after 8 to 12 weeks for the PHA/PLA blend fibers stored in soil. Such effects, which are—in principle—measurable, should be superimposed by the influence of the not completely homogeneous samples, which is indicated by the large standard deviation even in the unaged sample. We attribute the relatively large standard deviations in the measurement of tensile strength to the measurement on micrometer-sized individual fibers with individual defects, which will be leveled off in fiber bundles [54,55]. The decrease in the mean values of tensile strength during storage in soil should therefore be interpreted with caution.

Except for the PHA/PLA blend fibers buried in soil after 84 days, all fibers show no clear decrease in molecular weight (Figure 3b). On the contrary, there is rather an increase in molecular weight than a decrease, but, considering the relative error of molecular weight determination (~±10%), the molecular weight for PLA and PBS/PLA BICO fibers does not change markedly in soil burial duration during the 12 weeks of this test. PHA/PLA blend fibers buried in soil show a significant decrease after 84 days, which was not seen after 56 days and does not occur when the fibers are stored at ambient temperature and humidity but without soil. However, no evidence of biological attack can be clearly deduced from the electron microscope image of the PHA/PLA blend fibers in soil at ambient temperature.

**Figure 4:**
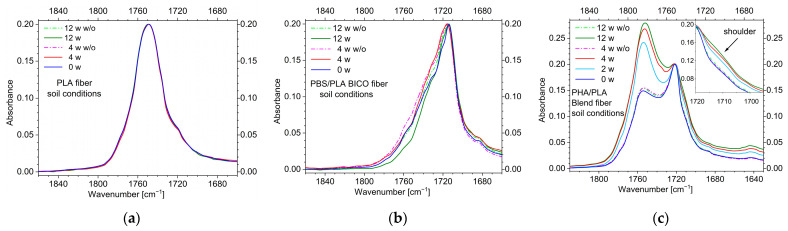
ATR-FT-IR spectra of the fiber surfaces in the carbonyl region of the (**a**) PLA fiber, (**b**) PBS/PLA BICO fiber, and (**c**) PHA/PLA blend fiber; dashed lines indicate storage under the same conditions but without (w/o) soil; spectra were baseline corrected and normalized to an absorbance of 0.2 at about 1750, 1717, or 1720 cm^−1^ (ν (C=O) PLA/PBS/PHA). No changes in the band contour are seen in the spectra of the PLA fiber, and only slight changes are seen in the spectra of the PBS/PLA BICO fiber. In contrast, the PHA/PLA blend fiber shows a clear enhancement of PLA or decrease of PHA, respectively. In addition, a shoulder in the short wavenumber range of the carbonyl band region can be seen when stored in soil but not when stored at ambient temperature without soil. Full ATR-FT-IR spectra are in supporting information, Figure S2.

The ATR-FT-infrared spectra of the three fibers in the carbonyl band area are shown in Figure 4. This surface-sensitive method clearly indicates no changes in the case of the PLA fibers. No additional bad or shoulder around 1700 cm^−1^, indicating free acid ends, can be seen. Therefore, significant hydrolysis of chains at the surface can be ruled out, and due to the similar shape of the spectra, there should be no changes in morphology such as changed proportions of the amorphous and crystalline phases.

The PBS/PLA BICO fiber also shows no clear evidence of acid terminations of the polymer chains in the carbonyl region (Figure 4b). However, due to the more structured contours of the bands in the short-wavelength range of the carbonyl band, changes in morphology should be present at the surface of the fibers.

In contrast, the PHA/PLA blend fiber shows a strong change in the band contour after a short time (Figure 4c). In relation to the intensity of the PHA-band at about 1720 cm^−1^, the PLA-band around 1755 cm^−1^ strongly increases. In the samples stored without soil, these changes are not seen, indicating the influence of the soil. In addition, additional intensity in the hydroxyl region around 3400 cm^−1^ with a tailing on the long-wavelength side can be seen in the case of the PHA/PLA blend fiber, indicating the presence of hydroxyl and acid functionalities (Figure S2, supporting information). Therefore, hydrolysis and degradation of PHA at the surface take place in soil but not in the samples stored without soil.

The DSC thermograms of the PLA fiber give no additional information, as they are nearly identical considering the manneristic selection of the baseline. No postcrystallization is detected in the first heating run, and the melting enthalpies are all in the range of 48 to 51 J g^−1^, indicating the highly crystalline nature of the melt spun PLA fiber. Crystallization in the cooling run is hindered (around 90 °C to 95 °C with ~1 J g^−1^ to 3 J g^−1^), and postcrystallization takes place in the second heating run at 110 °C to 120 °C with enthalpies of 31 J g^−1^ to 34 J g^−1^ and melting with enthalpies of 37 J g^−1^ to 43 J g^−1^ (Figure S1a and Table S1 supporting information).

**Figure 5:**
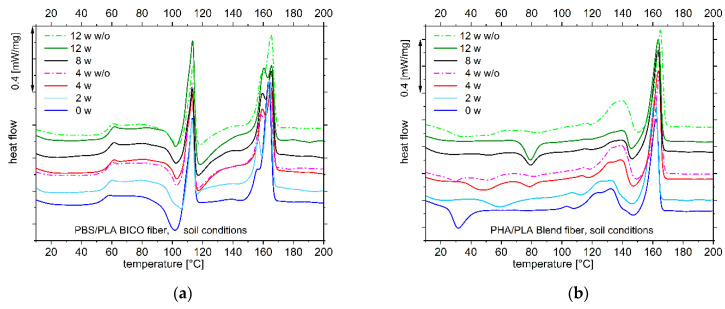
DSC thermograms of the second heating curve (10 K min^−1^) of the PBS/PLA BICO fiber (**a**) and PHA/PLA blend fiber (**b**) under soil conditions, showing changes in postcrystallization (both fibers) and melting of the PLA phase in the PBS/PLA BICO fiber; open symbols/dashed lines indicate storage under the same conditions but without (w/o) soil.

The DSC thermograms of the second heating of the PBS/PLA BICO fiber show an aging effect concerning the postcrystallization at around 100 °C and 120 °C: the enthalpy for the postcrystallization at 100 °C decreases and the enthalpy for the postcrystallization at 120 °C increases (Figure 5a; Table S3 supporting information).

Also, the shoulder at about 158 °C of the high melting peak around 162 °C in the unaged sample becomes more prominent. As this can be seen for both samples, either stored in soil or stored without soil at ambient temperature with the same humidity, and the samples were previously melted and cooled in a controlled manner, resolving the bicomponent core shell structure (second heating curve), these changes are not a surface effect. Obviously, the interaction of PBS and PLA can be changed by melting. Anyway, it should not be changed by storage; thus, the reason for changes in crystallization and melting behavior indicates changes in the chain topology during environmental aging at ambient temperature.

The DSC thermogram of the second heating of the PHA/PLA blend fiber also shows clear changes after storage in the soil (Figure S2b; Table S5 supporting information). Here, too, the postcrystallization temperature changes. While at the beginning a postcrystallization at about 30 °C is observed, which belongs to PHA [56,57], after 8 to 12 weeks a postcrystallization at 80 °C occurs, with intermediate stages apparent at 2 and 4 weeks. At the same time, the enthalpy of the melting peak at about 120 °C to 140 °C belonging to PHA [57,58,59,60] decreases. The samples that were not stored in soil do not show this change in enthalpy reduction but also exhibit altered postcrystallization behavior. Therefore, it is assumed that water sorption (and desorption) at ambient temperature has an impact on postcrystallization. The decrease in PHA and relative enhancement in PLA on the longer time scale seen in the FT-IR spectra indicate changes in the phase morphology of the blend. Therefore, the postcystallization at about 80 °C in the curves for 8 and 12-week storage presumably belongs to this “new” enriched PLA phase.

Since the IR spectrum gives evidence of the formation of carboxylic acids in the samples stored in soil but not in the samples stored only at ambient temperatures at the same humidity, a soil-mediated activity of hydrolysis and degradation must be postulated for the PHA/PLA blend fiber. Anyway, no obvious biological attack can be deduced from the SEM micrographs.

### 3.2. Compost/Elevated Temperature

#### 3.2.1. Compost: Microscopy

Under industrial composting conditions, the disintegration of organic material by biological attack does not proceed at ambient temperature. Even natural or homemade compost reaches elevated temperatures due to the activity of the microorganisms, which produce biomass and heat. In the case of several geotextiles, the soil will be heated by the sun, especially in areas inclined to sunlight. Testing the fibers at higher temperatures will give more insight into the behavior of the materials.

**Figure 6:**
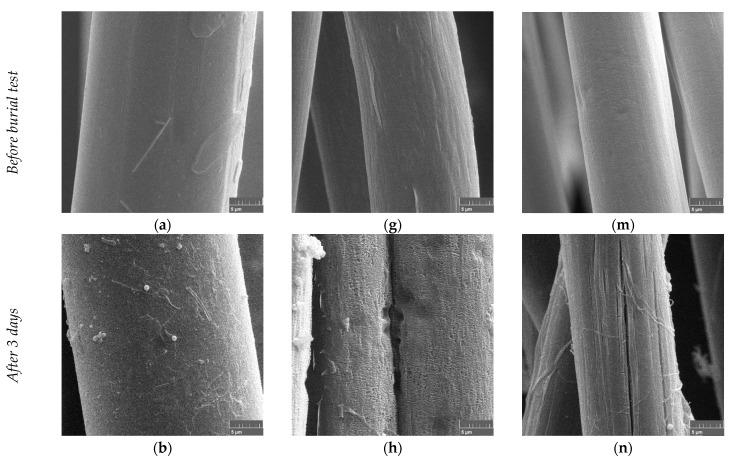

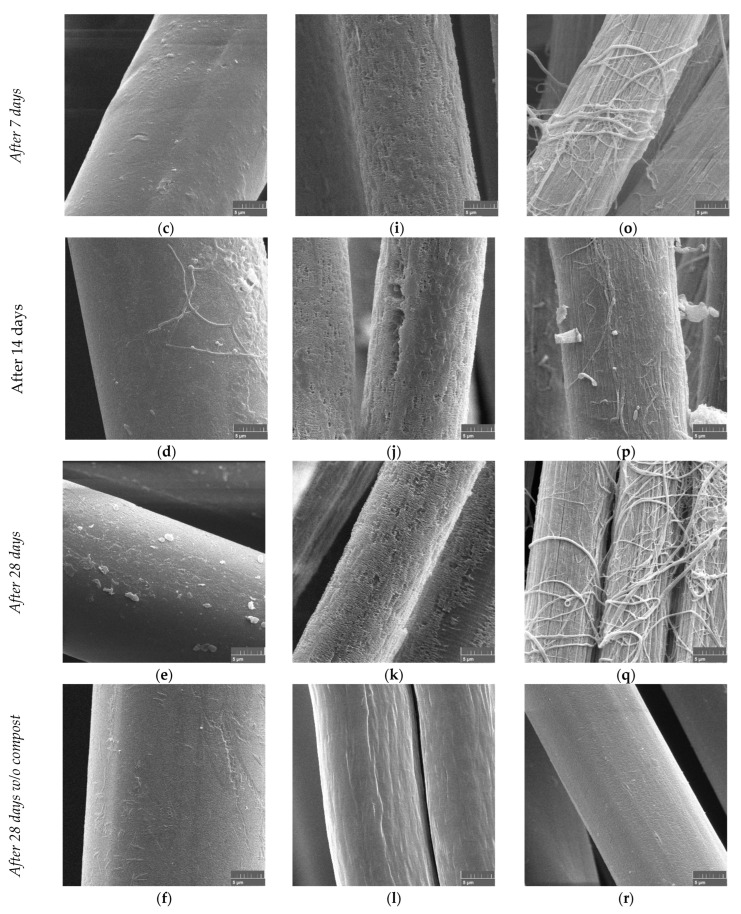
SEM micrographs of fibers buried in compost after 0, 3, 7, 14, and 28 days: (**a**–**f**) PLA, (**g**–**l**) PBS/PLA BICO fiber, and (**m**–**r**) PHA/PLA blend fiber; the size bar indicating 5 µm is placed at the bottom right of each image.

It is well known that PLA is not easily degraded by a biological attack below its glass transition point due to low mobility of the polymer chains slowing down hydrolytic degradation [61], ineffectiveness of enzymes in the crystalline and restricted amorphous (tie chains, free end chains, folding chains) regions of semicrystalline PLA, and missing proteins enhancing enzyme activity [62,63]. Therefore, it is somewhat surprising that surface erosion was detected in only one case of the fibers examined by electron microscopy (Figure 6d, detail enlargement, Figure S3 supporting information), albeit the patterns on the surface after three days (Figure 6b) can be interpreted as a fungal infestation, which is more clearly visible after 14 days. In general, the electron microscopic examinations of PLA fibers showed hardly any changes, contrary to the other fibers. This may be caused by the inappropriate microbiological composition of the used compost.

In contrast to the PLA fibers, the PBS/PLA BICO fibers show an eroded surface with hints of pitting after only three days. The pitting increases with longer storage, but there is no evidence of a microbiologic attack either. (Figure 6g–k). The sample stored at elevated temperature but without compost (Figure 6l) does not show the pattern of erosion of the surface even after 28 days, but some structuring of the surface can be seen. This may be due to the thermal and environmental stress of the PBS/PLA BICO fiber at 58 °C, which induces volume expansion due to the glass transition of the PLA core. This will result in mechanical stress at the surface of the skin, which may disintegrate into a pattern of crazes and microcracks supported by additional pressure build-up caused by water absorption. Furthermore, the surface destroyed and enlarged in this way may provide an improved attack for microorganisms but also for physical and chemical degradation mechanisms, so that hole formation may occur as a secondary effect (Figure 6j,k). Additional enzymatic breakdown of ester bonds due to bacteria—no fungal hyphae can be seen in Figure 6g–l—weakening the skin cannot be excluded.

Similar to the PHA/PLA blend fibers stored in the soil, cracking can be observed on the surface of the PHA/PLA blend fibers stored in compost after a short period of time (Figure 6n; three days). During further storage in compost, the surface of the fibers shows a pattern of fibrillation, but, due to the fiber architecture of the PHA/PLA blend fiber, this must be due to stresses. As this fiber is made from a “homogenous” blend mixture, relaxation due to thermal and environmental stresses should cause other patterns than in the case of a bicomponent fiber with a non-homogenous cross section. After seven days, there is evidence of microbial attack by fungi, as indicated by the tubular infestation with fungal hyphae of the fiber surfaces in some parts of the surface (Figure 6o,q). The fibers stored without compost but at elevated temperatures show no visible changes, which is somewhat surprising due to the thermal stresses as indicated above.

#### 3.2.2. Compost: Mechanics/Molecular Weight/FT-IR/DSC Characterization

**Figure 7:**
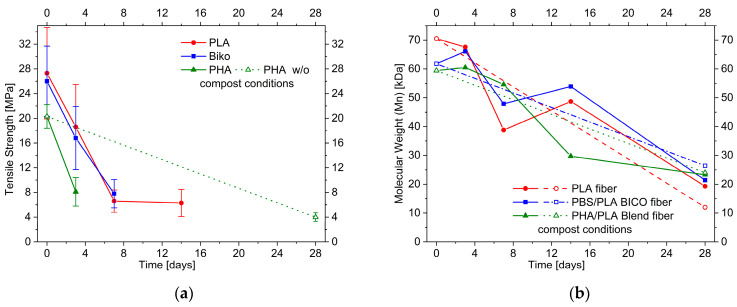
Effect of compost burial duration up to 84 days at 58 °C on the tensile strength (**a**,**b**) molecular weight (M_n_) of PLA, PBS/PLA BICO, and PHA/PLA blend fibers; open symbols indicate storage under the same conditions but without compost.

Storage in compost caused fibers to become brittle, so that tensile strengths could not be measured over the entire test period, as shown in Figure 7a. Although no degradation was optically visible in the PLA, the tensile strength decreased after a storage period of only three days to 85% of the initial value. After one and two weeks, the tensile strength decreases to 30% and 29%, respectively. After four weeks, tensile testing of the fibers stored in compost or without substrate in the climate chamber was no longer possible. The increasing brittleness of the fibers stored in the compost should reduce the influence of the individual fiber defects mentioned in Section 3.1.2 when stored in the soil. Therefore, the absolute standard deviations should become smaller when stored in compost, which is the case for PLA and PBS/PLA BICO fiber.

The tensile strength of the PBS/PLA BICO fibers decreased to 66% after three days and 30% after one week. After two weeks, no more measurements were possible. The fibers stored without substrate could not be tested after four weeks. For the PHA/PLA blend fibers, a measurement could only be made after three days. The tensile strength decreased by 40%. However, the comparative sample without substrate could be measured after four weeks. The tensile strength was 20% of the initial value.

The average molecular weight of the fibers decreased during storage in the climate chamber regardless of whether they had been in contact with compost, as shown in Figure 7b. After 28 days, the molecular weight of PLA was only 27% with compost contact, and 17% without compost contact compared to its original molecular weight. The PBS/PLA BICO fibers still had 37% and 43% of their original average molecular weight, respectively, while the PHA/PLA blend fibers had 39% and 40%, respectively. Thus, storage under composting conditions compared to storage at elevated temperatures did not result in a significantly higher reduction in molecular weight after 28 days. This degradation is a clear indication of chemical hydrolysis due to absorbed water. In addition, the PLA fibers are more susceptible to this chemical hydrolysis than the PBS/PLA BICO and the PHA/PLA blend fibers, which is likely due to their higher polarity. For the PLA fiber, the decrease in molecular weight to 27% or 17% after 28 days, respectively, corresponds to 2.7 or 4.9 bonds statistically hydrolyzed per polymer chain. With a starting molecular weight of about 70,000 Dalton this refers to about 0.3% or 0.5% of all bonds broken. Assuming a second-order kinetic equation with constant water content (Equations (1) and (2)):

(1) $$\frac{\partial \left[bond\right]}{\partial t}=-k\times {\left[H_{2}O\right]}_{const.}\times \left[bond\right]$$

(2) $$\ln\frac{{\left[bond\right]}_{t}}{{\left[bond\right]}_{0}}=-k\times {\left[H_{2}O\right]}_{const.}\times t$$

with about 0.5 mol/L water concentration absorbed [47], the following equation for calculating the reaction velocity constant k can be derived:

(3) $$2{\times t}^{-1}\times \ln\frac{{\left[bond\right]}_{0}}{{\left[bond\right]}_{t}}=k$$

This results in a velocity constant of about 8–14 × 10^−10^ L mol^−1^ s^−1^, which is of a reasonable order of magnitude for uncatalyzed hydrolysis of polyesters (Ethylacetat: 1.5 × 10 ^10^ L mol^−1^ s^−1^ at 25 °C [48]) taking the higher temperature (accelerating) and restriction due to the heterogenous solid state (hindered transition state, slowing down) reaction into consideration.

**Figure 8:**
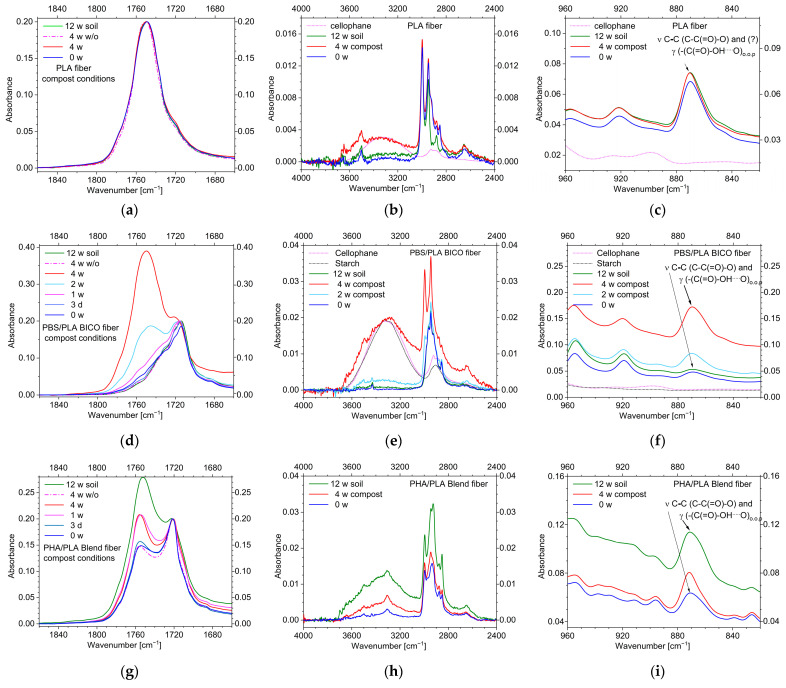
ATR-FT-IR spectra of the fiber surfaces buried in compost (in addition, the 12-week soil burial spectra are added for comparison); Spectra were baseline corrected and normalized to an absorbance of 0.2 at about 1750, 1717, or 1720 cm^−1^ (ν C=O PLA/PBS/PHA, respectively). (**a**–**c**) PLA fiber; (**d**–**f**) PBS/PLA BICO fiber; ((**g**–**i**) PHA/PLA blend fiber; in (**a**,**d**,**e**) the changes in the carbonyl region (ν C=O) are shown; in (**b**,**d**,**g**) the changes in the hydroxyl region ((ν O−H) are shown, including spectra of cellophane or starch for comparing the band contour of the fiber surfaces with polyhydroxylic carbohydrates; further on, in (**c**,**f**,**i**) the region of the deformation vibration of H-bridging hydroxyl groups (δ O−H− −O) is shown; no clear indication for carboxylic acid can be seen in the case of the PLA fiber (**a**–**c**). For the PBS/PLA BICO (**d**–**f**) and PHA/PLA blend fiber (**g**–**i**), the PLA carbonyl band at about 1750 cm^−1^ is enhanced in relation to the second polyester during storage under composting conditions. Under soil conditions the enhancement is more pronounced for the PHA/PLA blend fiber. The band contours in the hydroxyl regions indicate carboxylic acid formation for the PBS/PLA BICO and PHA/PLA blend fiber. Full ATR-FT-IR spectra can be found in the supporting information, Figure S5.

Unfortunately, no clear change in the contour of the carbonyl band can be seen even after 4 weeks at elevated temperature (Figure 8a) in the ATR-FT-IR spectra of the PLA fiber surface, which may be due to the low amount of carboxylic acid chain ends produced. In addition, the characteristic tailing of carboxylic acid hydroxyl group vibration at 3300 cm^−1^ to 2400 cm^−1^ [64] (Figure 8b) is too weak to give a clear indication. In addition, the characteristic out-of-plane deformation vibration of H-bridged carboxylic hydroxyl groups [64] is masked by the C-C vibration of the PLA chain [65] (Figure 8c).

Contrary to the PLA fiber, the ATR-FT-IR spectra of the surfaces of the PBS/PLA BICO fiber and the PHA/PLA blend fiber show strong changes in the carbonyl region (Figure 8d,g), indicating a loss of PBS or PHA at the surface, respectively. In the case of the PBS/PLA BICO fiber, the loss of PBS is remarkable after 2 weeks, in contrast to the 12-week storage in the soil with no remarkable change. In the case of the PHA/PLA blend fiber, the first deviations in the ATR-FT-IR spectra can be seen after three days, but, in contrast to the PBS/PLA BICO fiber, the change is more pronounced during 12-week storage in the soil.

The decrease in the intensity of the carbonyl band of PBS and PHA, respectively, is accompanied by an increase in the hydroxyl band as well as the out-of-plane vibration of the H-bridged carboxylic hydroxyl groups [64] (Figure 8e–g,i). This increase at approx. 875 cm^−1^ is so pronounced that in the case of the PBS/PLA BICO and PHA/PLA blend fibers, it must be regarded as a clear indication of acid-terminated chains.

The DSC thermograms of the second heating curve of the PLA, PBS/PLA BICO, and PHA/PLA blend fibers stored under compost conditions give no additional insight into morphological changes. No clear trend in post-crystallization can be seen for the PBS/PLA BICO fiber, while the corresponding thermograms of the PHA/PLA blend fiber confirm the trends described in the section presenting the results under soil conditions (Figures S1b and S4a,b, and Tables S2, S4 and S6 supporting information).

## 4. Discussion

Three different fibers for potential use in geotextiles were tested under different conditions. The fibers differed in terms of polymers and their properties, as well as morphology, and showed different responses to storage in soil or compost.

A surprising result of these tests is the relatively rapid loss of mechanical strength of the pure PLA fibers at elevated temperatures in the compost, although the fibers appear intact in microscopic observation and the surface ATR-FT-IR spectrum shows no significant changes. Since temperatures around 55 °C to 60 °C can also occur due to strong solar radiation, at least in the upper soil layers, it must be ensured that this loss of strength does not lead to a loss of functionality of a PLA geotextile. The rate constant for ester cleavage determined from the molar mass decomposition of PLA at these temperatures is in the order of magnitude of expected rates for uncatalyzed hydrolysis of ester molecules. Due to the small radius of the PLA fiber of about 13 μm, saturation with water occurs quickly. Assuming a diffusion constant of 10^−8^ cm^2^ s^−1^ [47] and constant occupation of the surface of the fiber with a film of moisture, diffusion is nearly complete in about two minutes [66], and equation 3 represents a good approximation. The somewhat higher velocity constant calculated for the fibers stored without compost but with the same humidity is straightforward, assuming the competition of compost or fiber absorbing the water, leading to lower activity of water. With a velocity constant of about 10^−9^ L mol^−1^ s^−1^ and assuming a velocity constant about two decades (~10^−11^ L mol^−1^ s^−1^) lower in soil at ambient temperature, during 12 weeks about 0.004% of bonds will be hydrolyzed, resulting in a drop in molecular weight of only about 3%, which is not measurable in GPC. Nine months will be needed for hydrolyzing about 0.01% of ester bonds, resulting in a drop in molecular weight of about 10 percent. Therefore, we can highlight that our observations concerning PLA in soil at ambient temperature indicate no significant influence of chemical hydrolysis.

The observations regarding the PLA fibers can also be applied to the PBS/PLA bi-component fibers for storage in soil. Here too, no significant changes were observed after 84 days in terms of strength, molecular weight, or detection of carboxylic acid ends. The changes in postcrystallization behavior measured in the DSC may be due to the anisotropic nature of the fibers, which generates new interfaces between the two polyesters during melting. These new interfaces can be sensitive to the presence of interfacially active components, such as even small, in the IR-spectrum, non-detectable amounts of acid-terminated chains, that can alter crystallization behavior.

In storage at ambient temperatures, the surface of the PBS/PLA BICO fiber does not change over the investigated period of 12 weeks. However, during storage in compost at an elevated temperature, deterioration of the surface of this fiber can be measured, and the surface structure is altered already after three days in compost. We interpret the roughened structure as an effect of swelling due to water absorption and volume expansion due to glass transition volume expansion of the PLA core, resulting in internal pressure. This internal pressure may be released radially, creating a rough pattern. We don’t have an explanation for the building up of the pits on the surface, as no bacterial colonization can be seen. Either the bacterial colonies were washed off with the compost from the fibers during cleaning or the already impaired surface was further destroyed mechanically by the brush used. Nevertheless, the observed decrease in PBS content in the BICO fiber during storage in compost in relation to the PLA amount can be interpreted as (i) a higher velocity of chemical hydrolysis of PBS in comparison to PLA, (ii) acid-catalyzed chemical hydrolysis of the outer shell, or (iii) biodegradation. Therefore, in further experiments, non-cleaned fibers should also be examined by SEM microscopy.

The PHA/PLA blend fiber is the only type that shows significant degradation in soil in our experimental series. The process is accompanied by minor bond cleavage after about 8 weeks, as observed in the IR spectrum and in GPC measurements. The higher intensity of the carboxylic acid-based hydroxyl band in soil than in compost at elevated temperatures is surprising. However, since polyhydroxyalkanoates as storage substances can be formed by many bacteria at ambient temperatures, they can also be degraded at lower temperatures, as the higher temperature in compost favors thermophilic bacteria.

The surface pattern of the PHA/PLA blend fiber in soil can be interpreted by the influence of adsorbed water, which builds up some internal pressure in the blend. This can result in the observed cracks on the weakened surface with PHA loss. Additionally, due to the elongation of the fiber in the manufacturing process, radial delaminations along the immiscible phases [56] of the blend fiber can be part of pressure relief. This assumption is supported by the surfaces of the samples buried in compost, which exhibit significantly stronger fibrillation. We interpret the tube-like infestation in Figure 6o,q, which also shows thickening in some areas, as fungal hyphae that were not brushed away during cleaning. Alternatively or additionally, fibrous fibrillations, which may be interwoven with the fungal material, can be considered.

## 5. Conclusions and Outlook

The results of our study show that for the selected substrates and conditions, the end of service life for micron-sized fibers is quickly reached due to environmental aging. As described by Lucas et al. among others, the environmentally induced aging of biodegradable materials is a complex process of physicochemical and biochemical processes [67]. With micron-sized fibers, it was to be expected that the various processes would occur in shorter periods of time, or rather, in parallel, than with specimens that have dimensions significantly larger than the micrometer scale. Mechanical strength decreased rapidly in the tested fibers at elevated temperatures, while biodegradation could not be detected with certainty via SEM. Measurements across the cross-section of the sample showing changes in the molecular structure, e.g., using Raman microscopy or confocal microscopy with single photon counting, could provide more accurate information on changes at the surface, just below the surface, and in the bulk of the sample, so that biotic and abiotic contributions can be separated, if necessary. The final biodegradation by assimilation of molecular fragments in the cell and mineralization is dependent on the precedent decomposition of the material to the size of molecular fragments. The process of this decomposition can be biotic through the activities of enzymes and active substances secreted by the microorganisms or abiotic through physicochemical processes. Especially in the case of polyesters, with their ester bonds that are easily hydrolysable compared to polyamides [48,68], and their importance in the plastics market, abiotic processes should always be taken into account in environmental aging studies over several months. The PHA/PLA blend fibers we tested at elevated temperatures broke down into fragments after only a short time due to predominantly abiotic processes caused by environmental conditions and ended their service life.

In materials science, it is common to describe the aging of materials to the time of product failure, not until ultimate degradation, in order to define the service life. Therefore, accelerated aging experiments are performed and evaluated using physicochemical approaches and models according to internationally recognized standards. Biotic contributions such as enzymatically enhanced hydrolysis follow completely different kinetic laws, and they are largely dependent on the microbial community and concentration in the environment. Therefore, aging tests using only classical service-life approaches are not useful for environmental aging tests, e.g., in the determination of the service life time and the degradation time up to digestible molecular fragments of biodegradable geotextiles.

In this study, we have shown that physicochemical processes have a great influence on the use of fine fibers in the environment, for example, as geotextiles. Mechanical properties may deteriorate in a short time, and physicochemical processes should not be neglected in the description of environmental aging. Further on, the changes in morphology due to physical processes caused by water absorption (and desorption) must be taken into account, as surface changes and enlargements can influence the subsequent processes. From our point of view, studies on aging in the environment should use as many analytical methods as possible. We believe that microscopic images should be taken of the cleaned samples for a good visualization of the fiber surface, but also of the uncleaned samples or of samples that have only been weakly cleaned using low forces to identify microbial colonization. Surface and profile examinations using spectroscopic methods can provide an insight into the physical changes of the material that is also important for the interpretation of the biochemical attack. The results might allow us to describe the changes from a kinetic point of view, in order to get better insights into the processes involved, depending on the environmental conditions.

However, the quality of the soil and compost used in the experiments must also be considered. Usually, as well as in our experiments, they are not optimized for biodegradable plastics as carbon sources. In order to make investigations of the aging behavior of biodegradable materials more comparable, standardized inocula would be useful, as Guo et al. [69,70] discussed in their studies on the relative rate of biodegradation using different inocula. Standardization could make it possible to generate basic metabolic kinetics after sufficient experimental data have been collected.

Especially in the case of non-optimal microbiological conditions and slowly biodegradable materials, the competing reaction of degradation by physicochemical effects must be considered to describe environmental aging comprehensively. The abiotic release of molecular fragments can be an important support for natural biodegradation.

## Data Availability

Not applicable.

## Supplementary Materials

The following supporting information can be downloaded at: https://www.mdpi.com/article/10.3390/polym15132959/s1, Tables S1–S6: DSC thermochemical data, Figure S1: (a,b) DSC thermograms of PLA, Figure S2: (a–c) ATR-FT-IR spectra of the fiber surfaces buried in soil, Figure S3: Detail enlargement of SEM micrographs of PLA, Figure S4: (a,b) DSC thermograms of fibers buried in compost, Figure S5: (a–c) ATR-FT-IR spectra of the fiber surfaces buried in compost.
