# Supplementary material for: Degradation Behavior of Biodegradable Man-Made Fibers in Natural Soil and in Compost
Source: Polymers (Basel). 2023 Jul 6;15(13):2959. doi: 10.3390/polym15132959 (PMC10347158; doi:10.3390/polym15132959)
Supplement: Supplementary file 1 [file polymers-15-02959-s001.zip › polymers-2358205-supplementary.pdf]

polymers

MDPI

Supporting information

Degradation Behavior of Biodegradable Man-Made Fibers in Natural Soil and in Compost

Pia Borelbach [1,*], Rodion Kopitzky [1,*], Jörg Dahringer [2] and Patrick Gutmann [2]

[1] Fraunhofer Institut für Umwelt-, Sicherheits- und Energietechnik UMSICHT, 46047 Oberhausen, Germany
[2] Indorama Ventures Fibers Germany GmbH, 86399 Bobingen, Germany
* Correspondence: pia.borelbach@umsicht.fraunhofer.de (P.B.); rodion.kopitzky@umsicht.fraunhofer.de (R.K.); Tel.: +49-(0)208-8598-1265 (P.B.); +49-(0)208-8598-1267 (R.K.)

Table S1. DSC thermochemical data of the PLA fiber in natural soil (30-200 °C; Isotherm 3 min, 200-0 °C, Isotherm 15 min, 0-250 °C, 10 K min^{-1}).

Soil conditions		cooling curve				first heating curve				
		T_g		T_c	ΔH_c	T_{cc}	ΔH_{cc}	T_m		ΔH_m
		°C	Cp J g^{-1} K^{-1}	°C	J g^{-1}	°C	J g^{-1}	°C		J g^{-1}
PLA	0 w	53.0	0.37	94.5	3.3			156.0	167.0	51
PLA	2 w	54.8	0.45	94.7	1.1	~140	0.5	156.0	168.5	48
PLA	4 w	51.3	0.49	97.2	1.3			156.0	164.3	51
PLA	8 w	53.9	0.47	94.2	2.1			157.0	164.3	50
PLA	12 w	54.5	0.48	94.2/116.3	0.6/1.3				164.8	51
PLA	4 w w/o	55.4	0,50	93.9	1.0				164.8	50
PLA	12 w w/o	53.8	0.51	91.8	1.3			156.0	164.9	49

Table S1. continued

Soil conditions		second heating curve						
		T_g		T_{cc}	ΔH_{cc}	T_m		ΔH_m
		°C	Cp J g^{-1} K^{-1}	°C	J g^{-1}	°C		J g^{-1}
PLA	0 w	57.0	0.46	106.0	35	157.2	166.0	40
PLA	2 w	58.9	0.48	120.4	33		166.0	37
PLA	4 w	58.9	0.48	121.3	33		163.4	38
PLA	8 w	59.1	0.50	109.5	33	159.2	165.8	43
PLA	12 w	58.5	0.50	120.2	33		164.3	43
PLA	4 w w/o	58.9	0.48	121.2	34		164.2	40
PLA	12 w w/o	89.3	0.47	121.0	33		164.9	38

Table S2. DSC thermochemical data of the PLA fiber in compost (30-200 °C; Isotherm 3 min, 200-0 °C, Isotherm 15 min, 0-250 °C, 10 K min−1).

Compost conditions	cooling curve				first heating curve					remarks
	T_g		T_c	ΔH_c	T_{cc}	ΔH_{cc}	T_m		ΔH_m	
	°C	Cp J g^{-1} K^{-}	°C	J g^{-1}	°C	J g^{-1}	°C		J g^{-1}	
PLA 0 w	53.0	0.37	94.5	3.3			156.0	167.0	51	
PLA 3 d	55.7	0.51	~94 br	1.0	~130	0.5	157.2	164.6	52	(cool) sh 123 °C
PLA 1 w	54.7	0.48	98.3	3.8			159.0	163.9	53	(cool) sh 119 °C
PLA 2 w	50.9	0.39	94.6	2.6			159.7	165.4	43	(cool) sh 119 °C
PLA 4 w	51.7	0,40	95.9	6.0			158.4	162.6	57	
PLA 4 w w/o	49.6	0.31	93.2	7.9			158.4		61	(cool) sh 102 °C, T_g 27 °C/ 0.14 J g^{-1}K^{-}

Table S2. continued

Compost conditions	second heating curve						
	T_g		T_{cc}	ΔH_{cc}	T_m		ΔH_m
	°C	Cp J g^{-1} K^{-1}	°C	J g^{-1}	°C		J g^{-1}
PLA 0 w	57.0	0.46	106.0	31	157.2	166.0	38
PLA 3 d	58.9	0.48	109.5	38	162.0	166.0	43
PLA 1 w	57.4	0.52	110.2	43	160,0	163.4	51
PLA 2 w	58.5	0,40	112.0	30	160.9	165.8	40
PLA 4 w	56.1	0.43	108.7	40	157.8	163.8	49
PLA 4 w w/o	52.0	0.50	105.2	40	153.2	159.9	56

Table S3. DSC thermochemical data of the PBS/PLA BICO fiber in natural soil (30-200 °C; Isotherm 3 min, 200-0 °C, Isotherm 15 min, 0-250 °C 10 K min^{-1}).

Soil conditions	cooling curve						first heating curve				
	T_g		T_c	ΔH_c	T_c	ΔH_c	T_m	ΔH_m	T_m	T_m	ΔH_m
	°C	Cp J g^{-1} K^{-1}	°C	J g^{-1}	°C	J g^{-1}	°C	J g^{-1}	°C	°C	J g^{-1}
BICO 0 w			77.5	16	105	7	115.4	20	155.9	164.2	36
BICO 2 w			78.8	20	106	1	114.7	22	155.7	163.8	33
BICO 4 w			75.8	18	138	2	115.1	22	157.1	163.0	34
BICO 8 w			79.7	16	106	1	114.8	21	155.9	163.6	35
BICO 12 w	54.3	0.3	79.6	23			114.7	27	155.9	163.1	34
BICO 4 w w/o			70.3	20			114.9	25	156.0	163.4	35
BICO 12 w w/o	53.0	0.21	79.4	16	106	3	115.3	21	sh	163.7	38

Table S3. continued

Soil conditions	second heating curve										
	T_g		T_{cc}	ΔH_{cc}	T_{cc}	ΔH_{cc}	T_m	ΔH_m	T_m	T_m	ΔH_m
	°C	Cp J g^{-1} K^{-1}	°C	J g^{-1}	°C	J g^{-1}	°C	J g^{-1}	°C		J g^{-1}
BICO 0 w	53.3	0.30	101.9	14	119.0	0.3	113.2	13	156.4	163.6	30
BICO 2 w	56.6	0.30	101.5	8	116.8	1	113.1	8	156.7	164.1	27
BICO 4 w	57.2	0.30	102.9	4	117.2	10	113.1	10	158.6	165.2	28
BICO 8 w	57.3	0.30	102.5	5	117.2	8	113.1	8	159.6	165.3	26
BICO 12 w	57.1	0.30	102.5	4	118.5	15	113.4	15	160.5	165.5	28
BICO 4 w w/o	56.2	0.30	102.9	6	117.2	9	113.1	11	158.6	164.6	28
BICO 12 w w/o	56.5	0.34	102.8	8	118.3	8	113.6	8	sh	165.2	29

Table S4. DSC thermochemical data of the PBS/PLA BICO fiber in compost (30-200 °C; Isotherm 3 min, 200-0 °C, Isotherm 15 min, 0-250 °C, 10 K min^{-1}).

Compost conditions	cooling curve						first heating curve					remarks
	T_g		T_c	ΔH_c	T_c	ΔH_c	T_m	ΔH_m	T_m	T_m	ΔH_m	
	°C	Cp J g^{-1} K^{-1}	°C	J g^{-1}	°C	J g^{-1}	°C	J g^{-1}	°C	°C	J g^{-1}	
BICO 0 w			77.5	16	105	7	115.4	20	155.9	164.2	36	
BICO 3 d	~53	0.24	79.4	19	105	3	114.4	21	156.2	163.5	35	
BICO 1 w	~50	0.12	75.0	17	102	9	115.1	22	157.1	163.0	34	
BICO 2 w	~49	0.14	79.7	14	101	5	113.6	16	157.3	164.3	39	(1st) 81.8 °C, 1.2 J g^{-1}
BICO 4 w	54.3	0,30	69.8	9	99	21	113.0	14	157.6	163.5	44	(1st) 157.6 > 163.5 °C
BICO 4 w w/o	53.0	0.21	79.4	16	106	3	115.3	21	sh	163.7	38	(1st) 81. 9 °C, 1.0 J g^{-1}

Table S4. continued

Compost conditions		second heating curve										
		T_g		T_{cc}	ΔH_{cc}	T_{cc}	ΔH_{cc}	T_m	ΔH_m	T_m	T_m	ΔH_m
		°C	Cp J g^{-1} K^{-1}	°C	J g^{-1}	°C	J g^{-1}	°C	J g^{-1}	°C		J g^{-1}
BICO	0 w	53.3	0.30	101.9	14	119.0	0.3	113.2	13	156.4	163.6	30
BICO	3 d	57.0	0.27	101.8	5	117.2	7	113.3	11	158.9	165.0	29
BICO	1 w	55.7	0.30	103.1	16	118.3	2	113.7	9	158.4	164.9	37
BICO	2 w	56.0	0.28	99.6	12	117.7	4	113.8	5	158.5	164.9	33
BICO	4 w	53.2	0.21	96.1	12	119.5	0.3	113.5	10	sh156	163.3	45
BICO	4 w w/o	56.5	0.34	102.8	8	118.3	8	113.6	8	sh	165.2	29

Table S5. DSC thermochemical data of the PHA/PLA blend fiber in soil (30-200 °C; Isotherm 3 min., 200-0 °C, Isotherm 15 min., 0-250 °C, 10 K min^{-1}).

Soil conditions				cooling curve				first heating curve					
		T_g		T_c	ΔH_c	T_c	ΔH_c	T_c	ΔH_c	T_m	ΔH_m	T_m	ΔH_m
		°C	Cp J g^{-1} K^{-1}	°C	J g^{-1}	°C	J g^{-1}	°C	J g^{-1}	°C	J g^{-1}	**°C**	J g^{-1}
PHA/PLA	0 w					87.6	18	120.9	2	137.3	22	166.7	29
PHA/PLA	2 w					88.1	20	122.8	2	136.4	19	165.4	31
PHA/PLA	4 w					86.1	18	127.2	1	139.4	18	164.7	33
PHA/PLA	8 w	~45	0.10			85.1	11	129.1	2	140.3	13	164.6	32
PHA/PLA	12 w	~45	0.14			84.2	10	128.2	2	140.7	12	165.8	34
PHA/PLA	4 w w/o			35.8	6	88.9	15	126.0	2	138.0	23	164.8	30
PHA/PLA	12 w w/o			34.8	5	90.6	12	126.4	2	138.5	23	165.4	30

Table S5. continued

Soil conditions		second heating curve										remarks
		T_{cc}	ΔH_{cc}	T_{cc}	ΔH_{cc}	T_{cc}	ΔH_{cc}	T_m	ΔH_m	T_m	ΔH_m	
		°C	J g^{-1}	°C	J g^{-1}	°C	J g^{-1}	°C	J g^{-1}	°C	J g^{-1}	
PHA/PLA	0 w	31.6	7			147.0	1	132.3	19	162.1	27	multi. peak 90-135: 103.1/120.9/132.3 °C
PHA/PLA	2 w	60.5	6			146.7	1	131.7	18	161.4	30	multi. peak 90-135: 106.2/125.6/131.7 °C
PHA/PLA	4 w	47.9	6	78.6	2	147.2	1	138.7	18	163.7	31	multi. peak 90-135: 113.3/130.1/138.7 °C
PHA/PLA	8 w	52.5	1	79.4	5	146.5	2	139.1	9	163.4	30	multi. peak 90-135: 115.2/132.7/139.1 °C
PHA/PLA	12 w			79.2	7	146.0	2	139.7	7	163.6	32	multi. peak 90-135: 115.6/131.7/139.7 °C
PHA/PLA	4 w w/o	29/51	9	81.6	2	149.6	1	138.5	25	164.5	26	multi. peak 90-135: 114.3/131.9/138.5 °C
PHA/PLA	12 w w/o	36/49	7	84.3	2	150.4	2	140.5	20	165.0	25	multi. peak 90-135: 115.5/133.0/140.5 °C

Table S6. DSC thermochemical data of the PHA/PLA blend fiber in compost (30-200 °C; Isotherm 3 min., 200-0 °C, Isotherm 15 min., 0-250 °C, 10 K min^{-1}).

Compost conditions		cooling curve				first heating curve			
		T_c	ΔH_c	T_c	ΔH_c	T_m	ΔH_m	T_m	ΔH_m
		°C	J g^{-1}	°C	J g^{-1}	°C	J g^{-1}	°C	°C
PHA/PLA	0 w	87.6	18	120.9	2	137.3	22	166.7	29
PHA/PLA	3 d	83.1	3	124.7	0	140.7	6	165.8	7
PHA/PLA	1 w	84.1	11	125.5	2	140,6	18	165.2	27
PHA/PLA	2 w	85.9	13	126.6	2	140.1	16	164.8	27
PHA/PLA	4 w	81.9	18	123.1	2	141.2	19	165.2	29
PHA/PLA	4 w w/o	86.4	17	123.0	2	137.2	22	164.4	26

Table S6. continued

Compost conditions	second heating curve										remarks
	T_{cc} °C	ΔH_{cc} J g^{-1}	T_{cc} °C	ΔH_{cc} J g^{-1}	T_{cc} °C	ΔH_{cc} J g^{-1}	T_m °C	ΔH_m J g^{-1}	T_m °C	ΔH_m J g^{-1}	
PHA/PLA 0 w	31.6	7			147.0	1	132.3	19	162.1	27	multi. peak 90-135: 103.1/120.9/132.3 °C
PHA/PLA 2 w	60.5	6			146.7	1	131.7	18	161.4	30	multi. peak 90-135: 106.2/125.6/131.7 °C
PHA/PLA 4 w	47.9	6	78.6	2	147.2	1	138.7	18	163.7	31	multi, peak 90-135: 113.3/130.1/138.7 °C
PHA/PLA 8 w	52.5	1	79.4	5	146.5	2	139.1	9	163.4	30	multi. peak 90-135: 115.2/132.7/139.1 °C
PHA/PLA 12 w			79.2	7	146.0	2	139.7	7	163.6	32	multi. peak 90-135: 115.6/131.7/139.7 °C
PHA/PLA 4 w w/o	29/51	9	81.6	2	149.6	1	138.5	25	164.5	26	multi. peak 90-135: 114.3/131.9/138.5 °C
PHA/PLA 12 w w/o	36/49	7	84.3	2	150.4	2	140.5	20	165.0	25	multi. peak 90-135: 115.5/133.0/140.5 °C

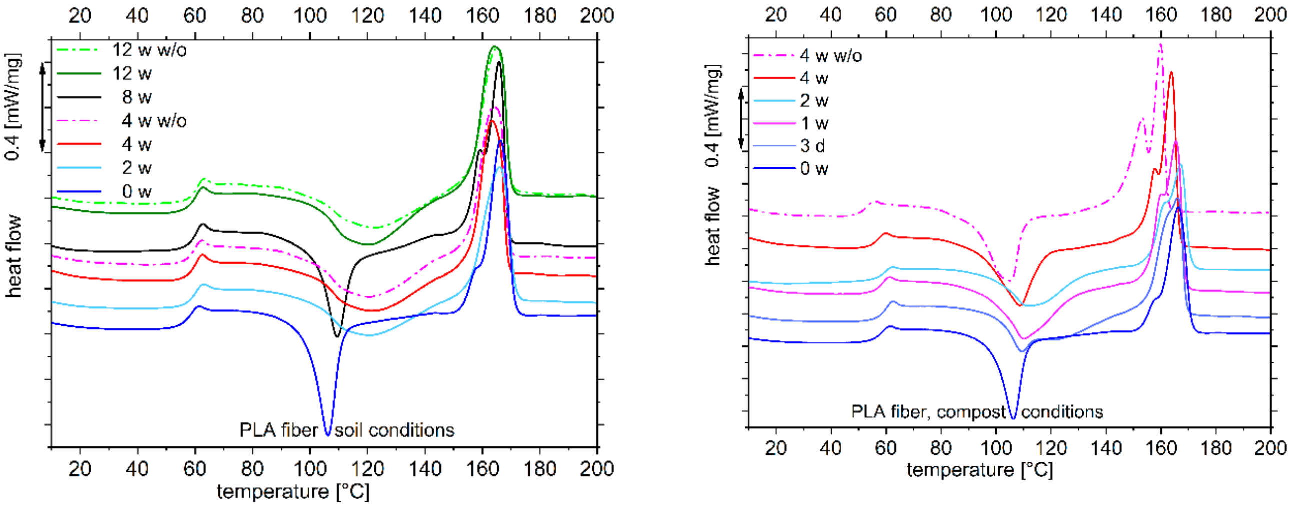

(a) (b)

Figure S1. DSC thermograms of the second heating curve (10 K min^{-1}) of the PLA in soil (**a**) and compost (**b**). In general, a shift to higher temperatures and a broadening of the post-crystallization peak occurs during storage at room temperature in both cases (with and without soil), and only a slight shift in the post-crystallization temperature occurs during storage at elevated temperature under compost conditions; the formation of two separate melt peaks is evident, which should belong to reorganization of imperfect crystals during storage at about glass transition temperature; open symbols, indicates storage under same conditions but without (w/o) soil or compost.

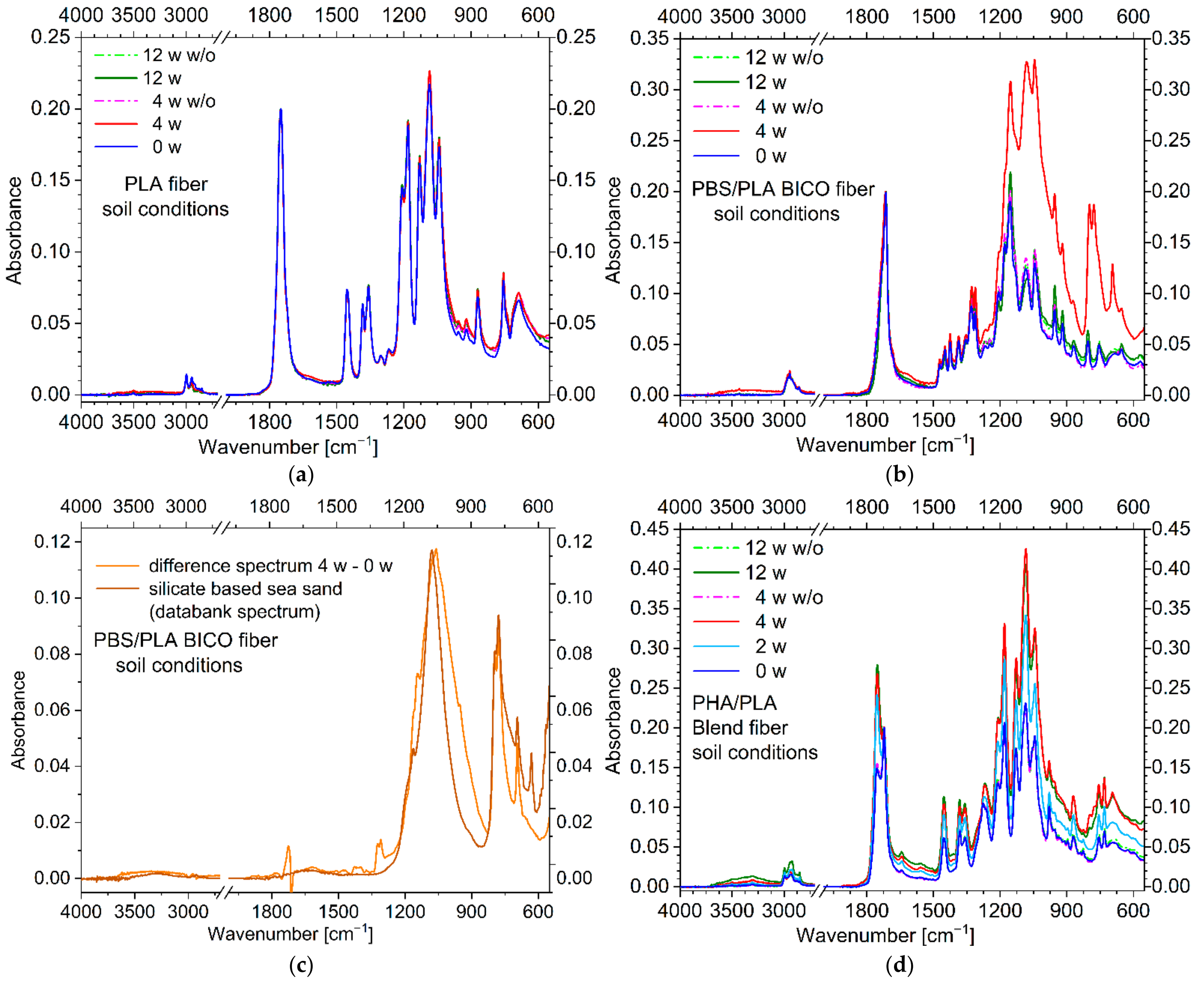

Figure S2. ATR-FT-IR spectra of the fiber surfaces buried in soil of the (**a**) PLA fiber, (**b**) PBS/PLA BICO fiber and (**c**) PHA/PLA blend fiber; dashed lines indicate storage under same conditions but without (w/o) soil; spectra were baseline corrected and normalized to an absorbance of 0.2 at about 1750, 1717 or 1720 cm^{-1} (ν (C=O) PLA/PBS/PHA). No changes in the band contour are seen in the spectrum of the PLA fiber (a) and only slightly changes are seen in the spectra of the PBS/PLA BICO fiber (**b**) with exception of the spectrum taken after 4 weeks in soil (red line). The strong enhancement in absorption below 1200 cm^{-1} should belong to a silicate based impurity at the surface of the measured fiber which was not wiped off during cleaning (see method section), as spectrum subtraction with the spectrum after 0 or 4 week results in a spectrum with strong intensity at around 1080 (very broad) and 780 cm^{-1}, which corresponds very well with a database spectrum of sea sand (**c**). In contrast, the PHA/PLA blend fiber (**d**) shows a clear enhancement of PLA or decrease of the PHA, respectively. Due to the normalization at 1720 cm^{-1} (ν (C=O) PHA) the bands of PLA in the fingerprint region also become more concise in the spectrum, resulting mainly in a PLA spectrum perturbed by some amounts of PHA after 12 weeks. In the C-H-region of the spectra, contour changes appear in particular in the sample after 12 weeks (~2920 / 2860 cm^{-1}), indicating a variable content of methylene groups. We interpret these changes with the presence of small amounts of process additives, which may accumulate on the surface with time and thus be measurable in the FT-IR-ATR technique.

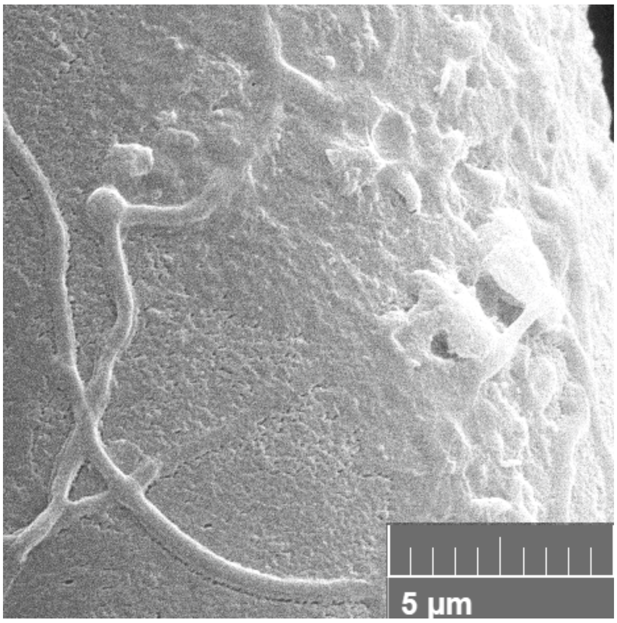

Figure S3. Detail enlargement of SEM micrographs of PLA fibers buried in compost after 14 days.

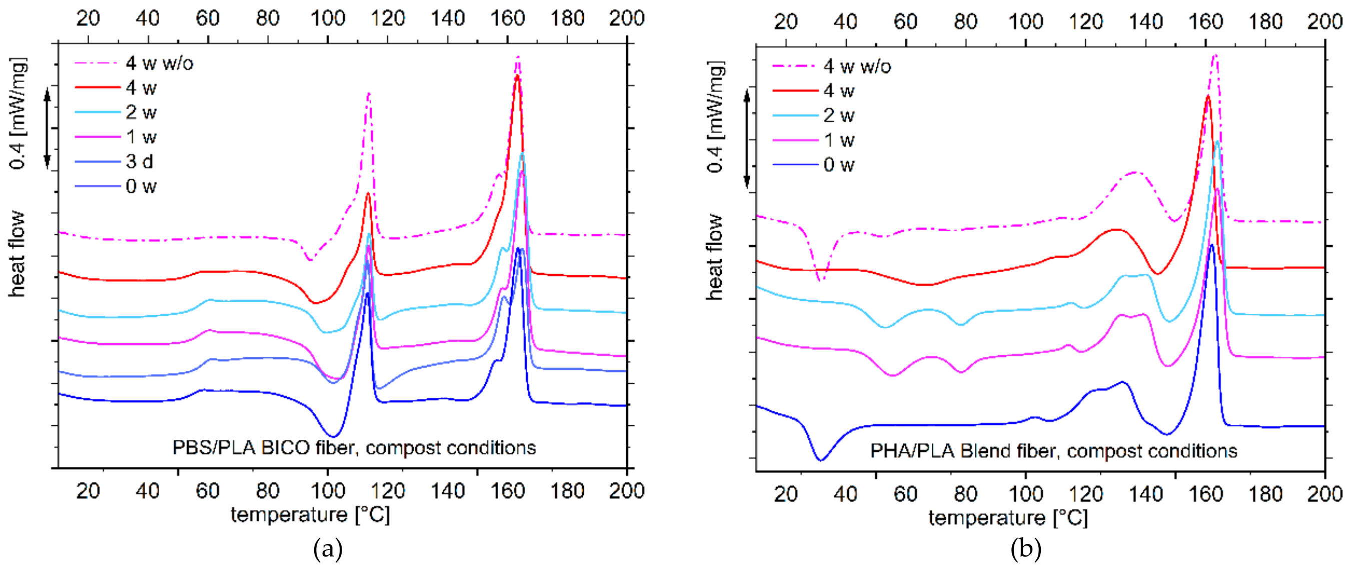

Figure S4. DSC thermograms of the second heating curve (10 K min^{-1}) of the PBS/PLA BICO fiber (**a**) and PHA/PLA Blend fiber (**b**) under compost conditions. Here the PHA/PLA Blend fiber show the same trend in postcrystallization as in the case of soil conditions, but no trend can be seen für the PBS/PLA BICO fiber; open symbols, indicates storage under same conditions but without (w/o) compost.

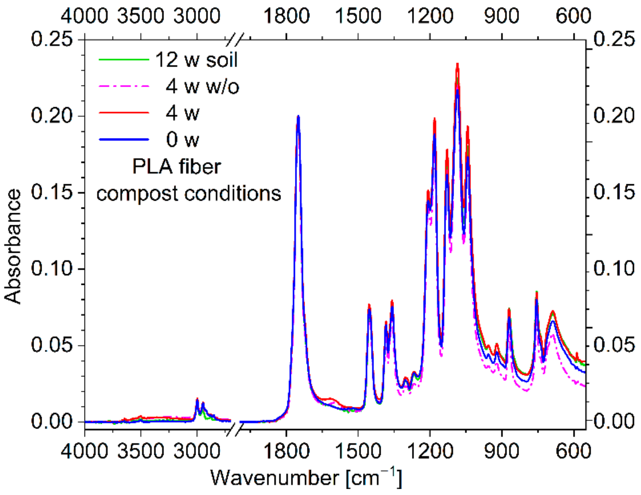

(**a**)

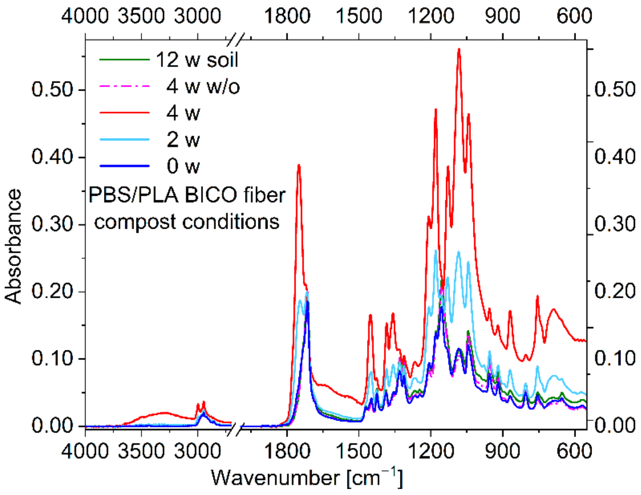

(**b**)

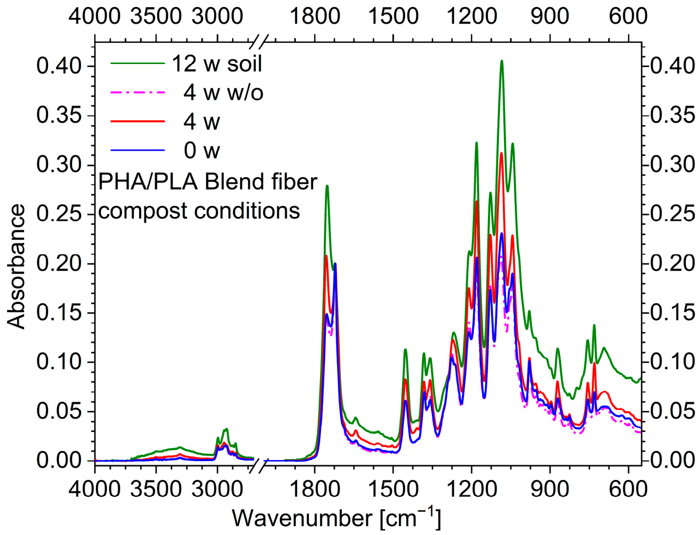

(**c**)

Figure S5. ATR-FT-IR spectra of the fiber surfaces buried in compost (in addition the 12-week soil burial spectra are added for comparison); Spectra were baseline corrected and normalized to an absorbance of 0.2 at about 1750, 1717 or 1720 cm^{-1} (ν C=O PLA/PBS/PHA, respectively). (**a**) PLA fiber; (**b**) PBS/PLA BICO fiber; due to the normalization, the bands of PLA in the fingerprint region also become more concise in the spectrum after 2 and 4 weeks, resulting in a PLA spectrum perturbed by some amounts of PBS at after 4 weeks in compost; (**c**) PHA/PLA blend fiber; due to the normalization at 1720 cm^{-1} (ν (C=O) PHA) the bands of PLA in the fingerprint region also become more concise in the spectrum after 2 and 4 weeks, resulting mainly in a PLA spectrum perturbed by some amounts of PHA after 4 weeks.
